# The comparison of evaluative effectiveness between antral follicle count/age ratio and ovarian response prediction index for the ovarian reserve and response functions in infertile women

**DOI:** 10.1097/MD.0000000000021979

**Published:** 2020-09-04

**Authors:** Shan-Jie Zhou, Ming-Jia Zhao, Cui Li, Xing Su

**Affiliations:** a Reproductive Medicine Center, Department of Gynecology and Obstetrics, Peking University International Hospital, Beijing; b Department of Reproduction and Genetics, Tangshan Maternity and Child Healthcare Hospital, Tangshan, China.

**Keywords:** anti-Müllerian hormone, antral follicle count, female, infertility, ovarian reserve, ovarian response prediction index, reproductive hormone

## Abstract

Aim of the present study was to explore the evaluative effectiveness of age, ovarian volume (OV), antral follicle count (AFC), serum follicle-stimulating hormone (FSH), anti-Müllerian hormone (AMH), AFC/Age ratio, AMH/Age ratio, FSH/luteinizing hormone (LH) ratio, and ovarian response prediction index (ORPI) to determine which could more advantageously assess ovarian reserve and response.

This research enrolled 319 consecutive infertile women who had undergone in vitro fertilization-ET/intracytoplasmic sperm injection (IVF-ET/ICSI) treatments. Abovementioned variables were measured and calculated. Receiver operating characteristic (ROC) curve analysis was used to analyze the predictive accuracy of variables and to calculate cut-off values and corresponding sensitivity and specificity.

Our study revealed that the significant variables for evaluating a decline in ovarian reserve include age, OV, FSH, AFC/Age ratio, AMH/Age ratio, and ORPI. Moreover, the area under the curve (AUC) of AFC/Age ratio was higher than other 5 variables (AUC = 1.000), and the cut-off value of AFC/Age ratio was 0.111 (sensitivity 100.00%, specificity 100.00%). The significant variables forecasting excessive ovarian response were age, AFC, AMH, FSH, AFC/Age ratio, AMH/Age ratio, FSH/LH ratio, and ORPI, and the significant variables forecasting poor ovarian response were AMH, LH, OV, AFC/Age ratio, AMH/Age ratio, and FSH/LH ratio. When ORPI was used to predict excessive response, the cut-off value of ORPI was 0.880 (sensitivity 84.72%, specificity 67.32%) and ORPI presented better effectiveness. When used to predict poor response, the evaluative effectiveness of 6 variables was almost similar, although the AUC of AFC/Age ratio presented the largest value.

Regarding the infertile women, AFC/Age ratio performed better than did the other variables in evaluating ovarian reserve, and it offered excellent effectiveness in predicting poor ovarian response, however, ORPI presented better effectiveness in predicting excessive ovarian response.

## 1 Introduction

Several studies^[1–4]^ have revealed that age, antral follicle count (AFC), and serum anti-Müllerian hormone (AMH) levels reflect the ovarian reserve admittedly, so these factors are considered valuable predictors of the ovarian response to exogenous gonadotrophins. In addition, AMH was a key for ovarian reserve-related outcomes, and has indeed been widely used in clinical practice. Sixty percent of the respondents from 796 infertility clinics worldwide reported using AMH as a first-line test in in vitro fertilization (IVF) cycles, and 54% reported AMH as the best test for evaluating ovarian reserve; 89% reported that AMH results were relevant to clinical practice.^[1]^ And AMH was an effective measure of quantitative ovarian reserve and was strongly associated with the ovarian response and oocyte yield after ovarian stimulation,^[2,3]^ moreover, using AMH, AFC, and age together constituted a new model for predicting poor or excessive ovarian response.^[4]^

However, several scholars have considered the evaluative effectiveness of the above parameters to be unsatisfactory and inaccurate for clinical practice. For example, Fleming et al^[5]^ stated that an ovarian reserve measure without limitations had not yet been discovered, although both AFC and AMH had good predictive value. Furthermore, debate exists regarding whether a single parameter or a combined index (follicle-stimulating hormone/luteinizing hormone ratio, FSH/LH ratio; ovarian response prediction index, ORPI) is a superior tool for evaluating the ovarian reserve or response. The FSH/LH ratio could be used to differentiate between decreased and normal response cycles, and the elevated day-3 FSH/LH ratio was associated with an inferior outcome in IVF treatment cycles.^[6–8]^ Oliveira et al^[9]^ innovatively used ORPI to assess ovarian response and found that ORPI exhibited an excellent ability to predict poor or excessive ovarian response, a collection of greater than or equal to 4 metaphase II oocytes and the occurrence of pregnancy in infertile women.

The objective of this study was to explore and compare the evaluative effectiveness of the abovementioned parameters and to determine which could be used to assess ovarian reserve and response advantageously.

## 2 Materials and methods

### 2.1 Subjects and study groups

We retrospectively enrolled 319 consecutive infertile women who had undergone IVF/intracytoplasmic sperm injection (ICSI) treatments from September 2016 to August 2017 in our fertility center. The infertile women aged 21 to 45 years had experienced infertility lasting 1 to 18 years, all subjects experienced at least 1 year without pregnancy success with natural attempts and were the first time to have IVF treatment, and administrated gonadotropin-releasing hormone (GnRH) antagonist protocol to perform ovulation induction. Patient history and clinical information were obtained from their medical records. All of the women were determined to have both ovaries present, no history of ovarian surgery, no severe endometriosis, and no evidence of endocrine disorders. Conventional IVF and/or ICSI were performed according to the cause of infertility. Infertile women were excluded if they were using fertility drugs (e.g., clomiphene, letrozole, and gonadotropin) or had any history of autoimmune and genetic disease, or iatrogenic conditions (e.g., radiation therapy or pelvic surgery), as these factors have been shown to alter the serum reproductive hormone and AMH levels. Patients were stratified into the following age groups: 21 to 29 years (Group 1), 30 to 34 years (Group 2), 35 to 39 years (Group 3), and 40 to 45 years (Group 4).

### 2.2 Measurement of reproductive hormones and AMH

Blood samples were obtained by venipuncture at 7:30 AM to 10:00 AM, and serum samples were measured together in the clinical laboratory of Peking University International Hospital. And the basal serum FSH, LH, estradiol (E2), total testosterone (TT) levels, and serum AMH levels were tested simultaneously on spontaneous cycle days 2–4 and prior to the beginning of IVF/ICSI cycles. The FSH, LH, E2, TT (kit from Abbott Ireland Diagnostics Division Lisnamuck, Longford Co., Longford, Ireland), and AMH (kit from Roche Diagnostics GmbH, Mannheim, Germany) levels were determined with commercial kits and an electrochemiluminescence immunoassay. The lower limits of the FSH, LH, E2, TT, and AMH levels were ≤10 pg/mL, 0.15 nmol/L, <0.05 IU/L, and ≤0.5 IU/L, and 0.010 ng/mL, respectively. The intra-assay coefficients of variation (CVs) for FSH, LH, E2, TT, and AMH were 4.6%, 5.1%, 6.7%, 2.3%, and 4.0%, respectively. The mean inter-assay CVs for FSH, LH, E2, TT, and AMH were 5.6%, 8.1%, 3.7%, 5.5%, and 2.7%, respectively.

### 2.3 Measurement of ovarian volume (OV) and antral follicle count

The experienced and qualified sonographers performed ultrasonographic evaluations for all subjects during spontaneous cycle days 2–4 using a two-dimensional transvaginal probe of 9 MHz frequency (HD11 XE, Philips Ultrasound, Inc., Bothell, WA). The total number of 2 to 9 mm antral follicles in both ovaries was measured and recorded. OV was calculated as the volume of an ellipsoid, that is, 0.52 × Length × Width × Depth. The total basal volume of both ovaries was evaluated in each patient.

### 2.4 Calculation of body mass index (BMI), AFC/Age, AMH/Age, FSH/LH ratio, and ORPI

Height and weight were measured by the investigators of our team and were used to calculate BMI, that is, weight (kg)/height (m^2^). Serum FSH, LH, and AMH concentration, AFC, and patient age were used to calculate FSH/LH ratio, AFC/Age ratio, AMH/Age ratio, and ORPI. The ORPI was defined by the following equation: ORPI = [AMH (ng/mL) × AFC (number)]/Patient age (years).^[9]^

### 2.5 Ovarian stimulation protocols

The ovarian stimulation protocols performed the GnRH antagonist protocols. The gonadotropin (Gn) was recombinant FSH (Gonal-f, Laboratoires Serono SA, Aubonne, Switzerland) and human menopausal gonadotrophin for injection (Livzon Pharmaceutical Group Inc. Zhuhai, Guangdong Province, China). The antagonist was Ganirelix Injection (ORGALUTRAN, Ravensburg, Germany). After the third day of treatment, the Gn dose was adjusted to the patient response. The total Gn dose was 2076.72 ± 963.33 IU, and the Gn treatment duration was 9.65 ± 2.74 days in each IVF/ICSI cycle. The trigger drug was recombinant human chorionic gonadotropin alpha for injection (OVIDREL 250 μg, Laboratoires Serono S.A. [LSA], Aubonne, Switzerland).

### 2.6 The definition of ovarian reserve, poor, and excessive ovarian response

The ovarian reserve was defined as women possessed the reproductive potential or the potential of oocyte yield at reproductive stage, and the biomarkers of ovarian reserve were being promoted as potential markers of reproductive potential or “fertility tests.” According to the criterion for the classification,^[9,10]^ the poor ovarian response was defined as collecting ≤3 oocytes, and the excessive ovarian response was defined as collecting ≥15 oocytes after the ovarian stimulation protocol.

### 2.7 Statistical analysis

Microsoft Excel 2013 software (Microsoft Corporation, Redmond, WA), SPSS 21.0 (IBM Corporation, New York, NY), and MedCalc Statistical Software version 18.2.1 (MedCalc Software bvba, Ostend, Belgium; http://www.medcalc.org; 2018) were used for all statistical analysis. The data were presented as the mean ± standard deviation (SD), as calculated for all subjects and each group. The one-way analysis of variance (ANOVA) test, multiple comparisons and multivariate analysis of variance were used to assess the differences between the mean values of parameters in the different groups. Pearson correlation analysis was used to assess the correlations between different parameters. Receiver operating characteristic curve (ROC curve) analysis was used to analyze the predictive accuracy of variables, and to calculate the area under the curve (AUC), and the cut-off values and corresponding sensitivity and specificity. The *Z* test was used to assess the differences between the AUC of different parameters. Tests were considered statistically significant if *P *<* *.05.

### 2.8 Ethics and informed consent statement

This study and the accompanying consent forms were approved by the Ethics Committee and Institutional Review Board of Peking University International Hospital, and Tangshan Maternity and Child Healthcare Hospital. Participants were enrolled in the study after written informed consent was obtained.

## 3 Results

### 3.1 One-way ANOVA analysis between different subgroups

**Figure 1:**
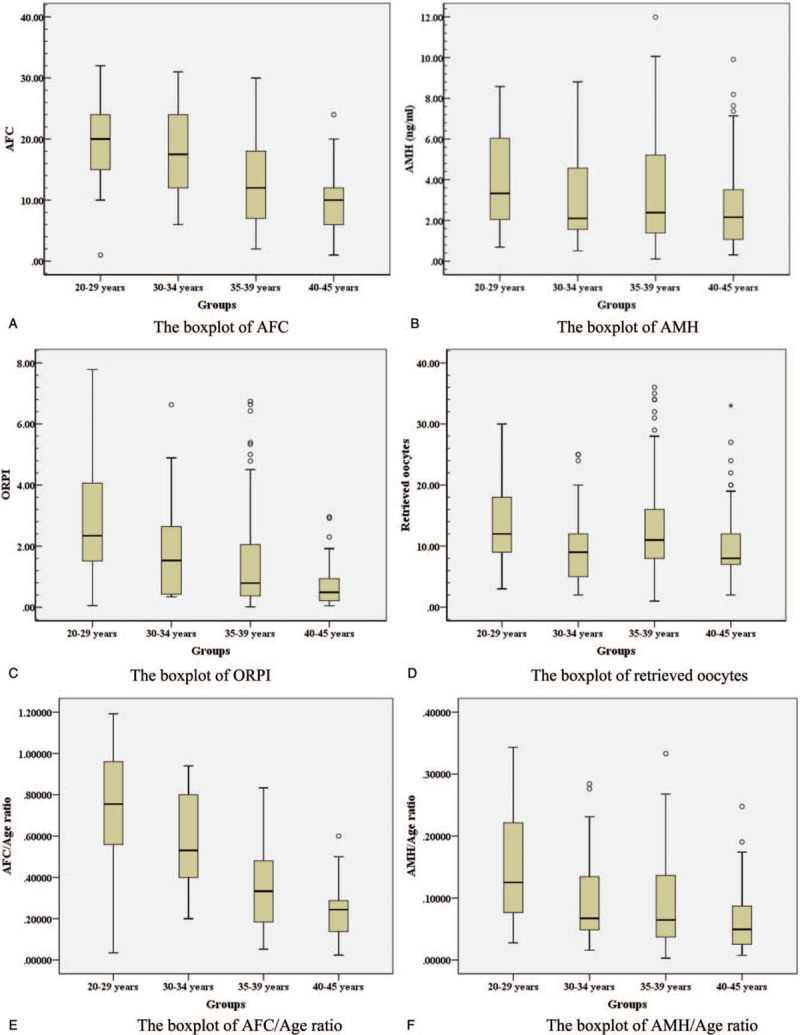
The boxplots of variables’ frequency distribution. AFC = antral follicle count; AMH = anti-Müllerian hormone; ORPI = ovarian response prediction index.

Clinical data and variable data of subjects presented in Table 1, and the boxplots of variables’ frequency distribution presented in Fig. 1A–F. According the diagnosis of patients, the rates of primary infertility type and secondary type were 37.04% and 62.96%, respectively. The rates of male factor, female factor, and double factor infertility were 7.41%, 25.93%, and 66.66%, respectively.

**Table 1:**
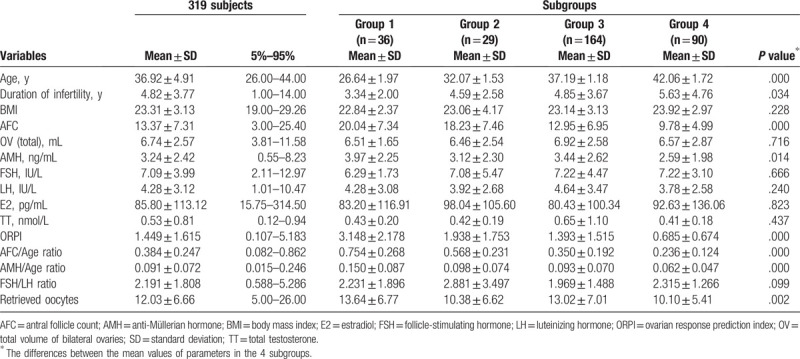
Frequencies of 319 subjects’ clinical data and variable data.

BMI, OV, serum FSH, LH, E2, TT, and FSH/LH ratio were not significantly different among the 4 subgroups; however, the duration of infertility, AFC, serum AMH levels, AFC/Age ratio, AMH/Age ratio, ORPI, and retrieved oocyte numbers were significantly different among the 4 subgroups.

Multiple comparisons showed that the durations of infertility were significantly different between Group 1 and Groups 3 and 4 (*P* = .034, *P* = .003). The AFC was significantly different between Group 1 and Groups 3 and 4 (*P* = .000, *P* = .000), between Group 2 and Groups 3 and 4 (*P* = .001, *P* = .000), and between Group 3 and Group 4 (*P* = .004). AFC decreased gradually as age increased, as presented in Fig. 1A. The AMH levels were significantly different between Group 1 and Group 4 (*P* = .004) and between Group 3 and Group 4 (*P* = .009). The AMH boxplot is presented in Fig. 1B. ORPI was significantly different between Group 1 and Groups 2, 3, and 4 (*P* = .005, *P* = .000, *P* = .000), between Group 2 and Group 4 (*P* = .001), and between Group 3 and Group 4 (*P* = .002). ORPI decreased gradually as age increased, as presented in Fig. 1C. The retrieved oocyte numbers were significantly different between Group 1 and Groups 2 and 4 (*P* = .046, *P* = .006), between Group 2 and Group 3 (*P* = .046), and between Group 3 and Group 4 (*P* = .001). The boxplot of retrieved oocyte numbers is presented in Fig. 1D. The AFC/Age ratio was significantly different between 4 subgroups (Group 1 versus Group 2: *P* = .001; *P* = .000 for others). AFC/Age ratio decreased gradually as age increased, as presented in Fig. 1E. The AMH/Age ratio was significantly different between Group 1 and Groups 2, 3, and 4 (*P* = .002, *P* = .000, *P* = .000), between Group 2 and Group 4 (*P* = .013), and between Group 3 and Group 4 (*P* = .001). The boxplot of AMH/Age ratio is presented in Fig. 1F.

### 3.2 Multivariate analysis of variance on ovarian reserve and response

According to multivariate analysis of variance, there was statistical significance in AFC (*P* = .000), OV (*P* = .001), and AFC/Age ratio (*P* = .002) for evaluating the ovarian reserve. And there was statistical significance in BMI (*P* = .010), FSH (*P* = .028), AFC, AMH, AFC/Age ratio, AMH/Age ratio, and ORPI (*P* = .000 for the latter 5 variables) for forecasting ovarian response.

### 3.3 Pearson correlation analysis

AFC demonstrated positive correlation with the OV (*P* = .009), AMH, AFC/Age ratio, AMH/Age ratio, and ORPI (*P* = .000 for latter 4 variables); however, AFC was negatively correlated with age (*P* = .000) and serum FSH level (*P* = .000). The above-mentioned parameters could reflect ovarian reserve.

There were positive correlations between the retrieved oocyte numbers and OV (*P* = .020), LH (*P* = .011), AFC, AMH, AFC/Age ratio, AMH/Age ratio, and ORPI (*P* = .000 for the latter 5 variables); however, a negative correlation existed between the retrieved oocyte numbers and age (*P* = .003). The above-mentioned parameters could reflect ovarian response.

There were negative correlations between age and AFC (*P* = .000), AMH (*P* = .018), AFC/Age ratio (*P* = .000), AMH/Age (*P* = .042), ORPI (*P* = .000), and retrieved oocyte numbers (*P* = .003). BMI was inversely correlated with FSH (*P* = .015) and FSH/LH ratio (*P* = .010) but did not correlate with AMH or AFC.

### 3.4 ROC curve analysis for evaluating ovarian reserve

**Figure 2:**
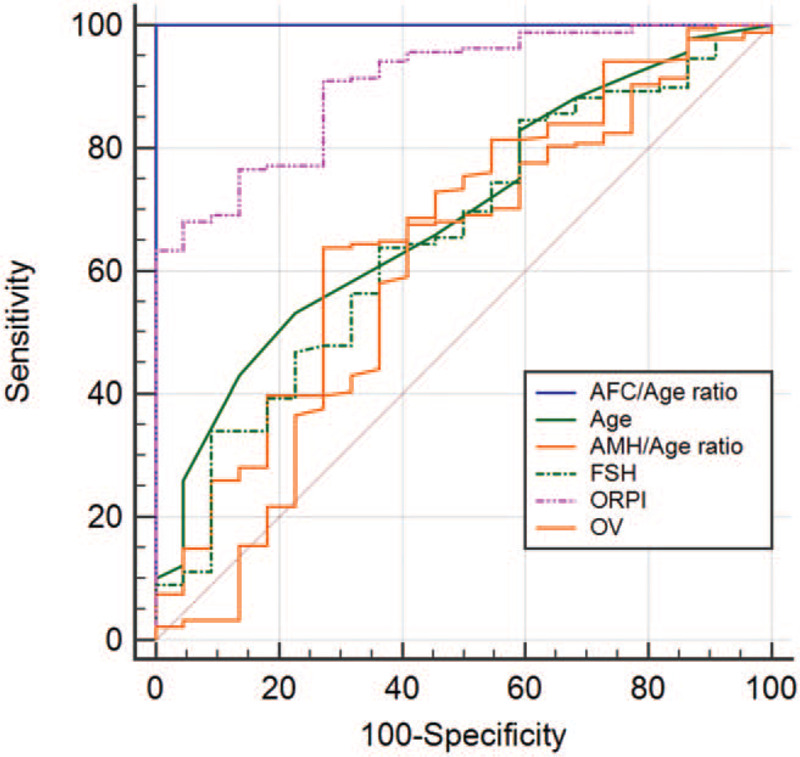
ROC curve of variables evaluating ovarian reserve decrease. AFC = antral follicle count; AMH = anti-Müllerian hormone; FSH = follicle-stimulating hormone; ORPI = ovarian response prediction index; OV = ovarian volume (total); ROC curve = receiver operating characteristic curve.

In general, AFC < 5 was one of the standards indicating decline of ovarian reserve.^[10]^ Using the abovementioned standards and the ROC curve to evaluate the significant variables of ovarian reserve decrease, the variables included AFC/Age ratio, AMH/Age ratio, age, OV, FSH, and ORPI, the corresponding AUC was 1.000, 0.641, 0.686, 0.641, 0.652, and 0.908, respectively. Although the AUC of AMH was 0.613, it was less valuable to access ovarian reserve (*P* = .0594). AUC, cut-off values, sensitivity, and specificity of variables evaluating ovarian reserve are presented in Table 2 and Fig. 2.

**Table 2:**
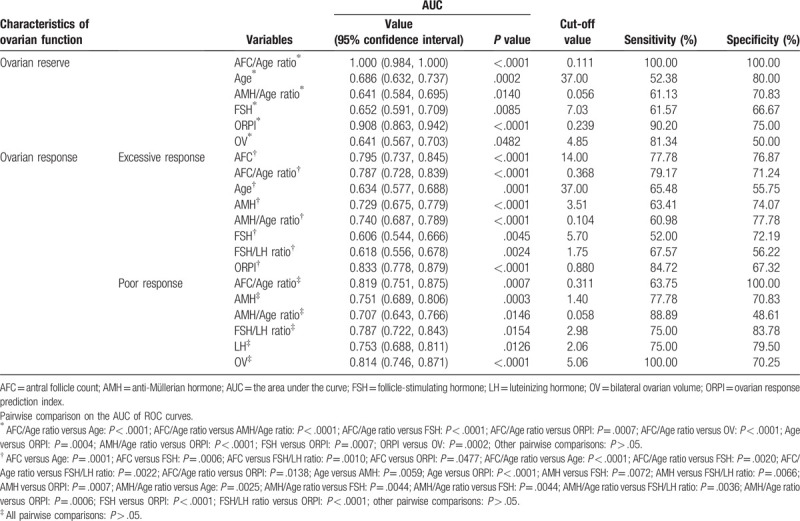
The AUC and cut-off values of variables evaluating ovarian reserve and response.

According to our results, ovarian reserve would decrease when subjects’ age and FSH were more than the cut-off values, moreover, the OV, AFC/Age ratio, AMH/Age ratio, and ORPI were less than the cut-off values.

### 3.5 ROC curve analysis for forecasting ovarian response

According to the retrieved oocyte numbers, ovarian response categories were divided into poor response (collecting oocytes ≤3), normal response (4–14 collecting oocytes) and excessive response (collecting oocytes ≥15).^[9,10]^

**Figure 3:**
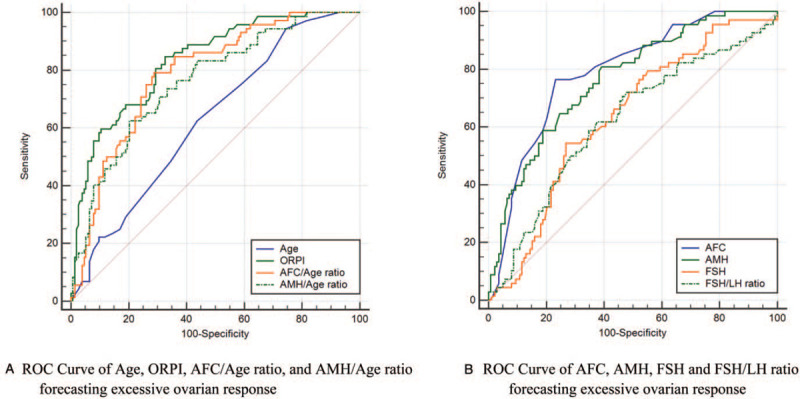
ROC curve of variables forecasting excessive ovarian response. AFC = antral follicle count; AMH = anti-Müllerian hormone; FSH = follicle-stimulating hormone; LH = luteinizing hormone; ORPI = ovarian response prediction index; ROC curve = receiver operating characteristic curve.

**Figure 4:**
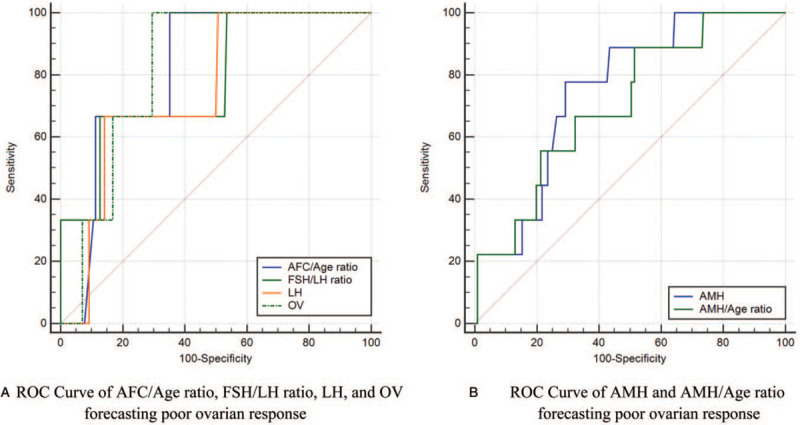
ROC curve of variables forecasting poor ovarian response. AFC = antral follicle count; AMH = anti-Müllerian hormone; FSH = follicle-stimulating hormone; LH = luteinizing hormone; OV = ovarian volume (total); ROC curve = receiver operating characteristic curve.

We used the abovementioned standards and the ROC curve to forecast the significant variables of excessive ovarian response, which included age, AFC, AMH, AFC/Age ratio, AMH/Age ratio, FSH, FSH/LH ratio, and ORPI, and to forecast the significant parameters of poor ovarian response, which included AMH, AFC/Age ratio, AMH/Age ratio, FSH/LH ratio, LH, and OV. The AUC, cut-off values, and corresponding sensitivity and specificity of variables evaluating ovarian response are presented in Table 2, Figs. 3A–B and 4A–B.

## 4 Discussion

Ovarian reserve can be altered or reduced due to age, disease, pelvic operation, chemotherapy, radiotherapy, and other risk factors, and it is beneficial to create treatment regimes, survey treatment effects, and forecast prognoses for infertile women by evaluating ovarian reserve or response. Our results indicated that the AFC, serum AMH levels, AFC/Age ratio, AMH/Age ratio, ORPI, and retrieved oocyte numbers were significantly different among the 4 subgroups and that there were negative correlations between age and AFC, AMH, AFC/Age ratio, AMH/Age ratio, ORPI, retrieved oocyte numbers. Moreover, we discovered that AFC, AFC/Age ratio, and ORPI decreased gradually with age. Lee et al^[11]^ and Raeissi et al^[12]^ observed increased FSH levels and decreased AMH levels with increasing age in women. Bozkurt et al^[13]^ reported that AMH was inversely correlated with age; however, AFC revealed a stronger correlation with age in both the fertile and infertile populations compared with basal FSH and AMH; the decrease in ovarian reserve in infertile patients was directly related to age, not infertility.

AFC on day 2–4 of the menstrual cycle, evaluated by transvaginal ultrasound, is commonly used to determine ovarian reserve, but AFC measurement is prone to error because of different sonographers. To explore the evaluative effectiveness of various variables, we analyzed the correlation of AFC with other variables and found that AFC showed a positive correlation with OV, AMH, AFC/Age ratio, AMH/Age ratio, and ORPI; AFC showed a negative correlation with age and serum FSH level. However, Somigliana et al^[14]^ insisted that low serum AMH is not associated with female subfertility.

The literature has reported that BMI is not associated with the AMH levels in the general population of infertile women or in patients without polycystic ovary syndrome (PCOS); however, BMI is significantly and inversely correlated with AMH in women with PCOS.^[15]^ Another study found that age is negatively correlated with AMH and AFC across all races (*P* < .05) and that elevated BMI is negatively correlated with AMH in Caucasian women but not in African-American, Hispanic, or Asian women.^[16]^ The results from our research showed that BMI was inversely correlated with FSH and FSH/LH ratio but did not correlate with AMH or AFC. In brief, debate still exists regarding the influence of BMI on ovarian function and AMH levels.

The ROC analysis results in our study revealed that the significant variables for evaluating ovarian reserve decrease included age, OV, FSH, AFC/Age ratio, AMH/Age ratio, and ORPI. Moreover, the AUC of AFC/Age ratio and ORPI was higher than those of the other 4 variables, and the diagnostic accuracy reached a “high” grade; the cut-off values of AFC/Age ratio and ORPI from ROC analysis were 0.111 (sensitivity 100.00%, specificity 100.00%) and 0.239 (sensitivity 90.20%, specificity 75.00%). Interestingly, the evaluative effectiveness of AFC/Age ratio exceeded that of ORPI (AUC: 1.000 vs 0.908, *P* = .0007). Assessments of the AMH and FSH levels in combination with female age could be helpful in predicting ovarian reserve in infertile women.^[12]^

Many studies^[17–21]^ have discussed how AFC and AMH could be used to assess ovarian reserve. However, our research did not find that these 2 variables acted as a single variable to significantly assess ovarian function. Serum AMH and AFC begin to decline in women between 34 and 35 years old, and AMH predicts biological age earlier than FSH or AFC do, and AFC does so earlier than FSH does.^[17]^ By age 32, over 50% of women with subfertility had AMH levels categorized as “low fertility” (AMH ≤19.5 pmol/liter), and the rate increased to 75% by age 39, with a decrease in mean AMH of 1.72 pmol/L/y.^[18]^ The serum AMH cut-off value for the normal ovarian reserve was calculated as 0.37 ng/mL (sensitivity 71.43%, specificity 66.67%, positive prediction 83.33%, negative prediction 50%).^[19]^ AMH should be considered a more reliable ovarian reserve assessment test compared with FSH because there was a strong positive correlation between the serum AMH level and AFC; further, the use of AMH combined with AFC may improve ovarian reserve evaluation.^[20]^ The present findings suggest the applicability of AMH determination as a marker for actual fertility in subfertile women with elevated basal FSH levels, as AMH was significantly associated with the timing of reproductive stages (i.e., the occurrence of menopausal transition or menopause during follow-up).^[21]^ Our results showed that the cut-off value of age was 37.00 for predicting ovarian reserve decline and that the corresponding sensitivity and specificity were 52.38% and 80.00%, respectively.

We found that the significant variables forecasting excessive ovarian response included age, AFC, AMH, AFC/Age ratio, AMH/Age ratio, ORPI, FSH, and FSH/LH ratio, and that the significant variables forecasting poor ovarian response included AMH, LH, OV, AFC/Age ratio, AMH/Age ratio, and FSH/LH ratio. Interestingly, ORPI and AFC/Age ratio demonstrated better effectiveness in evaluating ovarian response. When used to predict excessive response, the cut-off value of ORPI from ROC analysis was 0.880 (sensitivity 84.72%, specificity 67.32%). When used to predict poor response, the AUC of AFC/Age ratio presented the largest value despite no statistical difference among the 6 variables, the cut-off value of AFC/Age ratio was 0.311 (sensitivity 63.75%, specificity 100.00%). In addition, we found that it was inconsistent on the significant variables of evaluation between multivariate analysis of variance and ROC, such as AFC and BMI.

In recent years, many studies have focused on the value of a single parameter. For example, AMH was strongly associated with oocyte yield after ovarian stimulation and may therefore predicted ovarian response and the quality of oocytes and embryos.^[2,19,22]^ AMH and AFC had a higher predictive value for the responders than for FSH, E2, and chronological age, moreover, could predict the risk of ovarian hyperstimulation syndrome (OHSS) among patients.^[23,24]^ Vembu and Reddy^[25]^ reported ROC curve was plotted to predict the hyper response (OHSS), which showed a serum AMH cut-off value of 6.85 ng/mL with a sensitivity of 66.7% and a specificity of 68.7% for PCOS group and 4.85 ng/mL with a sensitivity of 85.7% and a specificity of 89.7% in non-PCOS group. AFC is superior to AMH in predicting poor ovarian response. The cut-off point for mean AMH and AFC in discriminating between poor and normal ovarian response cycles was 0.94 ng/mL (with a sensitivity of 70% and a specificity of 86%) and 5.5 (with a sensitivity of 91% and a specificity of 91%), respectively.^[26]^ Iranian women with a basal AMH level >6.95 ng/mL are at a high risk of developing OHSS, and those with AMH level <1.65 ng/mL are poor responders.^[27]^ Our results showed that the AMH cut-off value for excessive ovarian response and poor response was 3.51 ng/mL (sensitivity 63.41%, specificity 74.07%) and 1.40 ng/mL (sensitivity 77.78%, specificity 70.83%), AFC cut-off value for excessive ovarian response was 14.00 (sensitivity 77.78%, specificity 76.87%), respectively.

Currently, 2 popular combined indexes, ORPI and FSH/LH ratio, are used to assess ovarian function. Regarding the probability of collecting ≥4 oocytes, ORPI showed an AUC of 0.91 and an efficacy of 88% at a cut-off of 0.2, but for the probability of collecting ≥15 oocytes, ORPI showed an AUC of 0.89 and an efficacy of 82% at a cut-off of 0.9.^[9]^ The cut-off value reported by that study approximated our results. Oliveira and Franco^[28]^ reported the ORPI offered excellent ovarian response prediction (AUC = 0.91), and good predictions for the possibility of collecting >4 metaphase II oocytes (AUC = 0.84) and excessive ovarian response (AUC = 0.89) in infertile women, and ORPI value (≥1.7) was the benchmark that indicated high risk for OHSS. Selcuk et al^[29]^ found that the level of association between the ovarian response tests and poor ovarian response data was (in descending order): ovarian sensitivity index (OSI), ORPI, AFC, AMH, and age (AUC = 0.976, 0.905, 0.899, 0.864, 0.617, respectively), and OSI and ORPI could be superior to other ovarian responsiveness markers for poor and high ovarian responses on cycles with agonist or antagonist protocols. However, ORPI was more convenient than OSI, because OSI could be calculated after informed of the number of retrieved oocytes. In addition, opposing views on ORPI effectiveness continue to exist. Another study showed that both AMH and AFC were good predictors of ovarian response with an AUC >0.75 but that combining these variables was not necessary as ORPI would not improve the prediction value.^[30]^ Using the cut-off value derived from ROC analysis, cycles with an FSH/LH ratio ≥3 produced fewer mature oocytes (8.25 vs 11.74) and a higher percentage of poor ovarian response cycles (32.5% vs 14.3%). Additionally, the serum FSH level and FSH/LH ratio at the commencement of gonadotropin stimulation were inversely correlated to the number of mature oocytes.^[6]^ According to our results and previous reports in the literature, the abovementioned combined indexes had excellent performances in evaluating ovarian reserve and response.

Some shortcomings still exist in our research. First, there were no comparison data on ovarian function between fertile and infertile women. Second, we did not focus on predicting the influence of stimulation protocols and cycle cancellations. Previous research has shown that an elevated FSH/LH ratio >3 is more likely to result in the cancellation of the individual's cycle (15% vs 5.24%, *P* = .0001) and that the total gonadotropin dosage was greater in the higher-ratio group than in lower-ratio group (2636 vs 2242 IU; significant).^[31]^ Finally, we did not collect data on embryo quality and pregnancy outcome associated with parameters in this research. Several studies have paid close attention to treatment outcomes. An FSH/LH ratio <1.26 is associated with good oocyte parameters, high-quality embryos, and implantation after ICSI.^[32]^

## 5 Conclusions

Comparing the effectiveness of evaluating ovarian reserve and predicting ovarian response on age, OV, AFC, serum FSH, AMH, AFC/Age ratio, AMH/Age ratio, FSH/LH ratio, and ORPI, we found that AFC/Age ratio was superior to the other parameters in evaluating ovarian reserve, and it offered excellent effectiveness in predicting poor ovarian response, however, ORPI exceeded the other parameters in predicting excessive ovarian response. Consequently, we agreed that the evaluative effectiveness of a combined index exceeded that of a single parameter for evaluating the ovarian reserve and response of infertile women.

## Acknowledgments

The authors wish to thank all participants and their families for participating in this study. They gratefully acknowledge the help of Dr Ming-Jia Zhao, Dr Cui Li, and Dr Xing Su for the collection of data from Tangshan Maternity and Child Healthcare Hospital. And they also gratefully acknowledge the help of Dr Dian He for the statistical analysis, Dr He was from Department of Epidemiology and Health Statistics, School of Public Health, Capital Medical University, Beijing, China. They also thank Nature Research Editing Service from Springer Nature (http://www.springernature.com) for editing this manuscript.

## Author contributions

**Conceptualization:** Shan-Jie Zhou.

**Data curation:** Shan-Jie Zhou, Ming-Jia Zhao.

**Formal analysis:** Shan-Jie Zhou, Ming-Jia Zhao.

**Investigation & Measurement:** Shan-Jie Zhou, Ming-Jia Zhao, Cui Li, Xing Su.

**Methodology & Supervision:** Shan-Jie Zhou.

**Writing – original draft:** Shan-Jie Zhou, Ming-Jia Zhao.

**Writing – review & editing:** Shan-Jie Zhou.

## References

[1] Tobler KJ Shoham G Christianson MS Use of anti-mullerian hormone for testing ovarian reserve: a survey of 796 infertility clinics worldwide. J Assist Reprod Genet 2015;32:1441 8. 26347341 10.1007/s10815-015-0562-7 PMC4615913

[2] Peluso C Fonseca FL Rodart IF AMH: an ovarian reserve biomarker in assisted reproduction. Clin Chim Acta 2014;437:175 82. 25086280 10.1016/j.cca.2014.07.029

[3] Sahmay S Oncul M Tuten A Anti-müllerian hormone levels as a predictor of the pregnancy rate in women of advanced reproductive age. J Assist Reprod Genet 2014;31:1469 74. 25186502 10.1007/s10815-014-0324-y PMC4389927

[4] Brodin T Hadziosmanovic N Berglund L Comparing four ovarian reserve markers-associations with ovarian response and live births after assisted reproduction. Acta Obstet Gynecol Scand 2015;94:1056 63. 26184379 10.1111/aogs.12710

[5] Fleming R Seifer DB Frattarelli JL Assessing ovarian response: antral follicle count versus anti-Müllerian hormone. Reprod Biomed Online 2015;31:486 96. 26283017 10.1016/j.rbmo.2015.06.015

[6] Ho JY Guu HF Yi YC The serum follicle-stimulating hormone-to-luteinizing hormone ratio at the start of stimulation with gonadotropins after pituitary down-regulation is inversely correlated with a mature oocyte yield and can predict “low responders”. Fertil Steril 2005;83:883 8. 15820795 10.1016/j.fertnstert.2004.10.040

[7] Prasad S Gupta T Divya A Correlation of the Day 3 FSH/LH ratio and LH concentration in predicting IVF outcome. J Reprod Infertil 2013;14:23 8. 23926557 PMC3719363

[8] Seckin B Turkcapar F Ozaksit G Elevated day 3 FSH/LH ratio: a marker to predict IVF outcome in young and older women. J Assist Reprod Genet 2012;29:231 6. 22183503 10.1007/s10815-011-9695-5 PMC3288142

[9] Oliveira JB Baruffi RL Petersen CG A new ovarian response prediction index (ORPI): implications for individualised controlled ovarian stimulation. Reprod Biol Endocrinol 2012;10:94. 23171004 10.1186/1477-7827-10-94 PMC3566907

[10] Ferraretti AP La Marca A Fauser BC ESHRE consensus on the definition of ‘poor response’ to ovarian stimulation for in vitro fertilization: the Bologna criteria. Hum Reprod 2011;26:1616 24. 21505041 10.1093/humrep/der092

[11] Lee JY Jee BC Lee JR Age-related distributions of anti-Müllerian hormone level and anti-Müllerian hormone models. Acta Obstet Gynecol Scand 2012;91:970 5. 22574827 10.1111/j.1600-0412.2012.01448.x

[12] Raeissi A Torki A Moradi A Age-specific serum anti-mullerian hormone and follicle stimulating hormone concentrations in infertile Iranian women. Int J Fertil Steril 2015;9:27 32. 25918589 10.22074/ijfs.2015.4205 PMC4410034

[13] Bozkurt B Erdem M Mutlu MF Comparison of age-related changes in anti-Müllerian hormone levels and other ovarian reserve tests between healthy fertile and infertile population. Hum Fertil (Camb) 2016;19:192 8. 27499425 10.1080/14647273.2016.1217431

[14] Somigliana E Lattuada D Colciaghi B Serum anti-Müllerian hormone in subfertile women. Acta Obstet Gynecol Scand 2015;94:1307 12. 26332870 10.1111/aogs.12761

[15] Kriseman M Mills C Kovanci E Antimullerian hormone levels are inversely associated with body mass index (BMI) in women with polycystic ovary syndrome. J Assist Reprod Genet 2015;32:1313 6. 26238387 10.1007/s10815-015-0540-0 PMC4595400

[16] Moy V Jindal S Lieman H Obesity adversely affects serum anti-müllerian hormone (AMH) levels in Caucasian women. J Assist Reprod Genet 2015;32:1305 11. 26194744 10.1007/s10815-015-0538-7 PMC4595398

[17] Wiweko B Prawesti DM Hestiantoro A Chronological age vs biological age: an age-related normogram for antral follicle count, FSH and anti-Mullerian hormone. J Assist Reprod Genet 2013;30:1563 7. 23955628 10.1007/s10815-013-0083-1 PMC3843177

[18] Naasan MN Harrity C Pentony L Anti-Mullerian hormone normogram in an Irish subfertile population. Ir J Med Sci 2015;184:213 8. 24563261 10.1007/s11845-014-1089-0

[19] Celik E Bastu E Dural O Relevance of anti-Müllerian hormone on in vitro fertilization outcome. Clin Exp Obstet Gynecol 2013;40:66 9. 23724510

[20] Barbakadze L Kristesashvili J Khonelidze N The correlations of anti-mullerian hormone, follicle-stimulating hormone and antral follicle count in different age groups of infertile women. Int J Fertil Steril 2015;8:393 8. 25780521 10.22074/ijfs.2015.4179 PMC4355926

[21] Yarde F Voorhuis M Dólleman M Antimüllerian hormone as predictor of reproductive outcome in subfertile women with elevated basal follicle-stimulating hormone levels: a follow-up study. Fertil Steril 2013;100:831 8. 23755952 10.1016/j.fertnstert.2013.05.009

[22] Xu H Zeng L Yang R Retrospective cohort study: AMH is the best ovarian reserve markers in predicting ovarian response but has unfavorable value in predicting clinical pregnancy in GnRH antagonist protocol. Arch Gynecol Obstet 2017;295:763 70. 28012077 10.1007/s00404-016-4274-8

[23] Asada Y Morimoto Y Nakaoka Y Age-specific serum anti-Müllerian hormone concentration in Japanese women and its usefulness as a predictor of the ovarian response. Reprod Med Biol 2017;16:364 73. 29259490 10.1002/rmb2.12055 PMC5715898

[24] Jamil Z Fatima SS Cheema Z Assessment of ovarian reserve: anti-Mullerian hormone versus follicle stimulating hormone. J Res Med Sci 2016;21:100. 28163746 10.4103/1735-1995.193172 PMC5244648

[25] Vembu R Reddy NS Serum AMH level to predict the hyper response in women with PCOS and non-PCOS undergoing controlled ovarian stimulation in ART. J Hum Reprod Sci 2017;10:91 4. 28904496 10.4103/jhrs.JHRS_15_16 PMC5586096

[26] Mutlu MF Erdem M Erdem A Antral follicle count determines poor ovarian response better than anti-Müllerian hormone but age is the only predictor for live birth in in vitro fertilization cycles. J Assist Reprod Genet 2013;30:657 65. 23508679 10.1007/s10815-013-9975-3 PMC3663963

[27] Aghssa MM Tarafdari AM Tehraninejad ES Optimal cutoff value of basal anti-mullerian hormone in iranian infertile women for prediction of ovarian hyper-stimulation syndrome and poor response to stimulation. Reprod Health 2015;12:85. 26357853 10.1186/s12978-015-0053-4 PMC4565016

[28] Oliveira JB Franco JG Jr The ovarian response prediction index (ORPI) as a clinical internal quality control to prevent ovarian hyperstimualtion syndrome. JBRA Assist Reprod 2016;20:91 2. 27584597 10.5935/1518-0557.20160021 PMC5264369

[29] Selcuk S Bilgic BE Kilicci C Comparison of ovarian responsiveness tests with outcome of assisted reproductive technology - a retrospective analysis. Arch Med Sci 2018;14:851 9. 30002704 10.5114/aoms.2016.62447 PMC6040134

[30] Ashrafi M Hemat M Arabipoor A Predictive values of anti-müllerian hormone, antral follicle count and ovarian response prediction index (ORPI) for assisted reproductive technology outcomes. J Obstet Gynaecol 2017;37:82 8. 27976974 10.1080/01443615.2016.1225025

[31] Kofinas JD Elias RT Follicle-stimulating hormone/luteinizing hormone ratio as an independent predictor of response to controlled ovarian stimulation. Womens Health (Lond) 2014;10:505 9. 24807379 10.2217/whe.14.31

[32] Rehman R Syed H Iqbal NT FSH/LH ratio in females and intracytoplasmic sperm injection. J Pak Med Assoc 2015;65:1330 3. 26627517

